# Variation in Mycorrhizal Associations with Tulasnelloid Fungi among Populations of Five *Dactylorhiza* Species

**DOI:** 10.1371/journal.pone.0042212

**Published:** 2012-08-03

**Authors:** Hans Jacquemyn, Agnieszka Deja, Koen De hert, Bruno Cachapa Bailarote, Bart Lievens

**Affiliations:** 1 Division of Plant Ecology and Systematics, Biology Department, KU Leuven, Heverlee, Belgium; 2 Scientia Terrae Research Institute, Sint-Katelijne-Waver, Belgium; 3 Laboratory for Process Microbial Ecology and Bioinspirational Management, Lessius University College, Campus De Nayer, Consortium for Industrial Microbiology and Biotechnology (CIMB), Department of Microbial and Molecular Systems, KU Leuven Association, Sint-Katelijne-Waver, Belgium; University of Tartu, Estonia

## Abstract

**Background:**

Orchid species rely on mycorrhizal symbioses with fungi to complete their life cycle. Although there is mounting evidence that orchids can associate with several fungi from different clades or families, less is known about the actual geographic distribution of these fungi and how they are distributed across different orchid species within a genus.

**Methodology/Principal Findings:**

We investigated among-population variation in mycorrhizal associations in five species of the genus *Dactylorhiza* (*D. fuchsii*, *D. incarnata*, *D. maculata*, *D. majalis* and *D. praetermissa*) using culture-independent detection and identification techniques enabling simultaneous detection of multiple fungi in a single individual. Mycorrhizal specificity, determined as the number of fungal operational taxonomic units (OTUs), and phylogenetic diversity of fungi were compared between species, whereas discriminant analysis was used to compare mycorrhizal spectra across populations and species. Based on a 95% cut-off value in internal transcribed spacer (ITS) sequence similarity, a total of ten OTUs was identified belonging to three different clades within the Tulasnellaceae. Most OTUs were found in two or more *Dactylorhiza* species, and some of them were common and widespread, occurring in more than 50% of all sampled populations. Each orchid species associated with at least five different OTUs, whereas most individuals also associated with two or more fungal OTUs at the same time. Phylogenetic diversity, corrected for species richness, was not significantly different between species, confirming the generality of the observed orchid mycorrhizal associations.

**Conclusions/Significance:**

We found that the investigated species of the genus *Dactylorhiza* associated with a wide range of fungal OTUs from the Tulasnellaceae, some of which were widespread and common. These findings challenge the idea that orchid rarity is related to mycorrhizal specificity and fungal distribution.

## Introduction

The Orchidaceae is one of the most species-rich families within the Angiosperms, with an estimated number of >25 000 species [1]. Many orchid species have suffered dramatic declines in distribution and abundance, and at present several species have become rare or are threatened with extinction [2], [3]. In most cases, the decrease in abundance has been attributed to anthropogenic influences, including grazing, severe landscape modification and fragmentation, drainage or collection of wild species [2]–[4].

To maintain viable populations, most orchid species rely on two critical interactions: pollination by animals (mostly insects) and mycorrhizal symbioses with fungi [4]–[7]. Pollinators are needed to provide successful pollination and seed set, whereas mycorrhizal fungi provide the necessary resources needed to promote seed germination and seedling establishment [7]–. Any disturbance of these two interactions is likely to affect the population dynamics and long-term viability of orchid species, but their relative importance remains poorly understood. It is generally assumed that loss of mycorrhizal fungi has an immediate impact on the population dynamics of orchid species, particularly in short-lived species, whereas changes in pollinator diversity and/or abundance are likely to become visible only in the longer term [4].

The effects of anthropogenic disturbances on orchid viability through altered mycorrhizal associations are likely to depend on mycorrhizal specificity [7]. Additionally, these effects are also likely to depend on the geographic distribution of the mycorrhizal fungi. Orchid species that associate with a limited number of fungi or fungi with a narrow distribution area can be hypothesized to be more vulnerable to changes in mycorrhizal abundances than orchid species that associate with a large suite of mycorrhizal fungi or fungi with a very broad distribution [10]–[12]. Although convincing evidence is still lacking, recent analyses of mycorrhizal associations in the Australian genus *Caladenia* have shown that mycorrhizal associations combined with other environmental factors can have a strong influence on plant rarity [12]. On the other hand, no relationship was found between orchid rarity and mycorrhizal specificity in the genus *Drakaea*. However, because the formation of mycorrhiza was restricted to specific microhabitats, it was shown that mycorrhizal associations limited the abundance of *Drakaea* in some landscapes [13].

Despite the many studies that have investigated mycorrhizal associations in orchids, little is still known about the actual distribution of orchid mycorrhizal fungi [14], and few studies have investigated mycorrhizal association patterns across multiple populations within a single species. For example, it is not clear whether individuals from different populations of a single species associate with the same set of mycorrhizal fungi or whether this may vary from one site to the next, perhaps depending on environmental conditions. Several orchid species have been shown to use phylogenetically closely related fungal species [15]–[17], whereas in other species associations with several fungi are common [7], [18], [19]. Moreover, associations have been shown to vary within species among life cycle stages [20]–[23], and there is some evidence that some species may be able to switch to different fungi under adverse environmental conditions [24] or in different parts of their distribution range [18], [25], [26].

In this study, we investigated mycorrhizal associations in multiple populations of five species of the orchid genus *Dactylorhiza*. The mycorrhizal ecology of this genus is still poorly understood, and only few studies have investigated mycorrhizal associations in this genus [27], [28]. The main goal of this study was to identify the main pool of fungal species associating with the studied orchid species. More specifically, we investigated how mycorrhizal associations differed across different populations within a single orchid species, and whether results were consistent across different species. Moreover, by taking into account the phylogenetic relationships of the identified fungi, this analysis allowed comparison of the phylogenetic breadth among populations within species and among different species. Finally, we also tested the hypothesis that orchid rarity was affected by mycorrhizal specificity by relating the frequency of occurrence of the different orchid species to the number and phylogenetic diversity of fungal OTUs orchids associated with.

## Materials and Methods

### Ethics Statement

All necessary permits were obtained for the described field studies.

**Figure 1:**
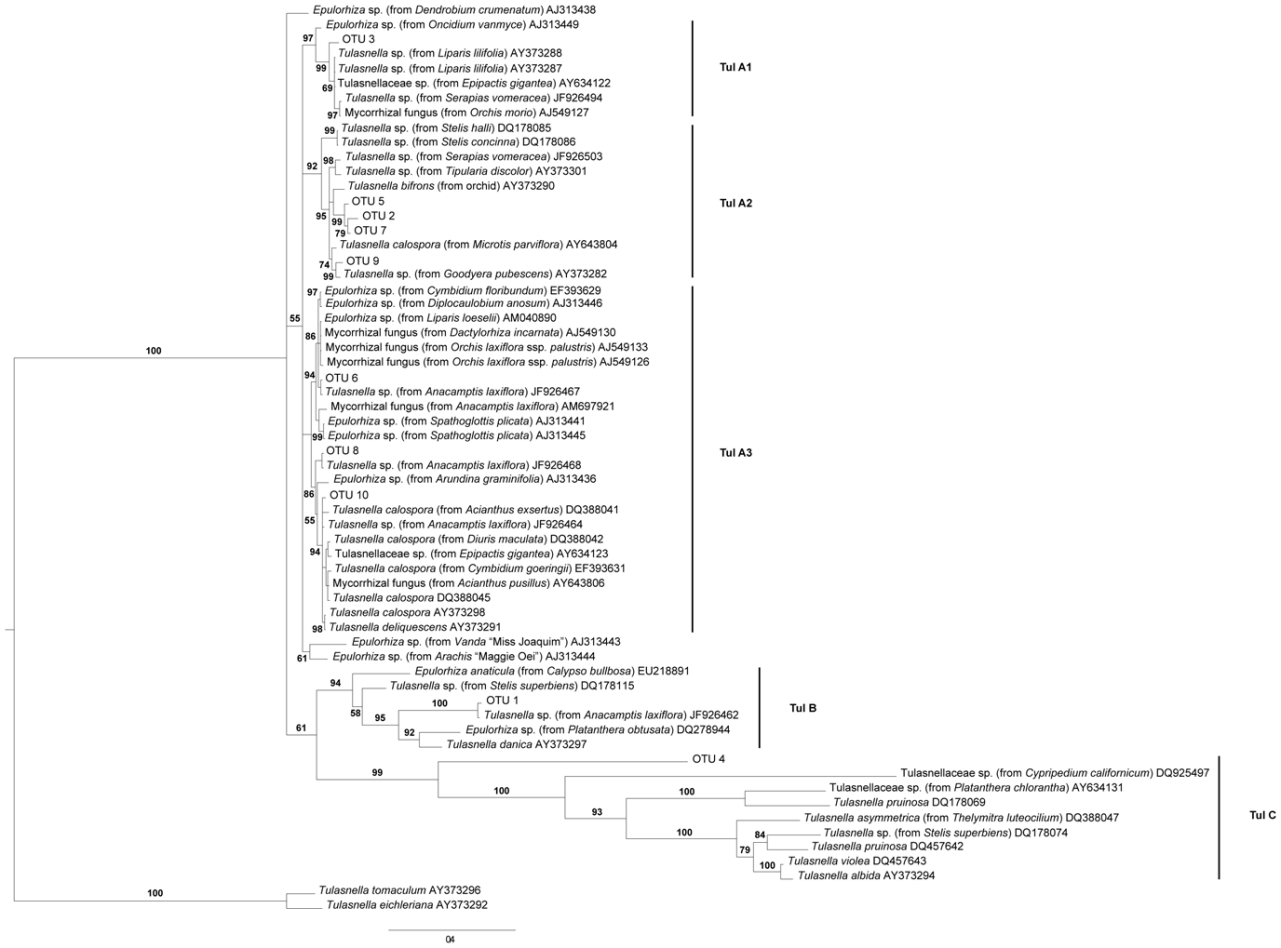
Bayesian majority consensus tree based on internal transcribed spacer (ITS) sequences of Tulasnellaceae fungi. The tree was computed under the GTR+G substitution model (5 000 000 generations run) and includes representatives of European, American, and Australian meadow and forest photosynthetic orchids, tropical terrestrial and epiphytic orchids, non-orchid species and fungal strains and fruitbodies. *Tulasnella tomaculum* and *Tulasnella eichleriana* were used as outgroup taxa. Branch support: Bayesian posterior probabilities (BPP).

### Study Species and Sampling

The genus *Dactylorhiza* consists of a large group of species that are widely distributed across the boreal and temperate zones of Europe, Asia, North America and Northern parts of Africa [29]. Its taxonomical status is quite complex due to high morphological variation of many taxa and the numerous intra- and inter-genus hybrids. Most *Dactylorhiza* species are summergreen. The leafy shoots appear in early spring and mostly last until autumn. In contrast to species of the genus *Orchis*, tubers are lobed or palmately divided. All species are mycorrhizal. Mycorrhizal colonization is mainly observed in the slender roots and sometimes in the extremities of the finger-like extensions of the tuber [30].

**Table 1:** List of fungal operational taxonomic units (OTUs)^a^ identified using cloning and sequencing.

OTU	Representativesequence^b^	Length (bp)	Phylogenetic relationship^c^	
			Closest match in GenBank (Accession no.)	Sequenceidentity (%)
**OTU1**	JX024729	728	Uncultured *Tulasnella* clone 1124a (FJ788890)	94
**OTU2**	JX024730	757	Uncultured *Tulasnella* mycobiont of *Aneura pinguis* clone 9573A (EU909346)	96
**OTU3**	JX024731	743	Uncultured *Tulasnella* mycobiont of *Aneura pinguis* clone 9764(EU909268)	99
**OTU4**	JX024732	705	*Tulasnella irregularis* isolate C3-DT-TC-2 (GU166423)	97
**OTU5**	JX024733	754	Uncultured *Tulasnella* mycobiont of *Aneura pinguis* clone 9570B(EU909337)	100
**OTU6**	JX024734	782	Uncultured *Tulasnella* clone 18tu-10 (HM230650)	97
**OTU7**	JX024735	753	Uncultured *Tulasnella* mycobiont of *Riccardia multifida* clone 9592B (EU909305)	98
**OTU8**	JX024736	745	*Tulasnella calospora* strain MAFF P305801 (DQ388041)	96
**OTU9**	JX024737	759	*Tulasnella* sp. 141 (AY373264)	98
**OTU10**	JX024738	735	*Tulasnella calospora* strain MAFF P305801 (DQ388041)	96

a Fungi were grouped into OTUs defined by 95% internal transcribed spacer (ITS) sequence similarity.

b GenBank accession number.

c Based on BLAST analysis (May 2011).

In the summers of 2007 and 2008, root samples were collected from a total of 126 individuals of five species of the genus *Dactylorhiza*, including *Dactylorhiza fuchsii*, *D. incarnata*, *D. majalis*, *D. maculata,* and *D. praetermissa*. The first three species are diploids (2*n* = 40), whereas *D. maculata* and *D. praetermissa* are tetraploids (2*n* = 80) [29]. Compared to most other orchid species in Belgium [2], these species have a relatively broad distribution. *D. maculata* is the most common species occupying 13.3% of 4×4 km grid squares in Belgium, whereas *D. incarnata* is the rarest species (2.2% of all grid squares). *D. majalis*, *D. praetermissa* and *D. fuchsii* occur in 10.8, 2.6, and 2.2% of all grid squares, respectively.

**Figure 2:**
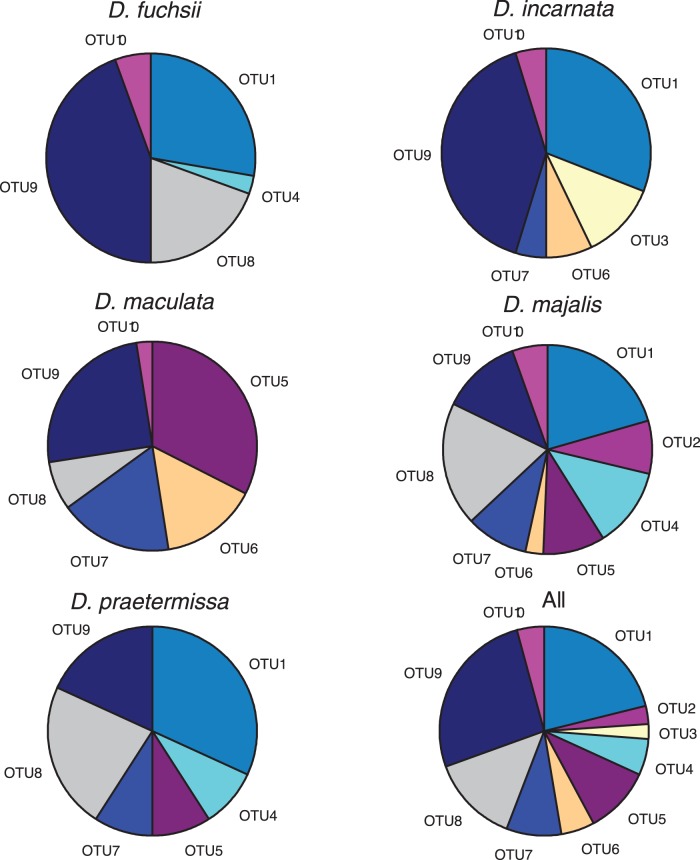
Frequency distribution of the ten observed operational taxonomic units (OTUs) among five *Dactylorhiza* species (a-e) and among all studied *Dactylorhiza* species together (f).

Samples were collected from 24 different sites that were distributed across Belgium (Fig. S1). Sites consisted of dune grasslands, wet meadows and open forests. In most populations, five individuals per population were sampled, but in some cases it was not possible to sample more than three individuals. The average sampling size was 4.6 individuals per population. For each individual, two root pieces were carefully excavated, placed in plastic bags and brought to the laboratory at 4°C for further analysis.

**Table 2:** The number of operational taxonomic units and phylogenetic diversity (PD) values for each sampled population of five *Dactylorhiza* species.

Species	Population	Numberof OTUs	PD_obs_	PD_null_
*D. fuchsii*	Hobokense Polder	3	0.8220	0.4158
	Ter Yde 1	2	0.6341	0.2787
	Torfbroek	3	0.8284	0.4336
	Baronville	3	0.8284	0.4457
	Han-Sur-Lesse	4	1.1048	0.5513
*D. incarnata*	Westhoek	3	0.8220	0.4144
	Oude Landen	2	0.7581	0.2721
	Ter Yde 2	4	0.8454	0.5561
	Ekers Moeras	3	0.8197	0.4292
	Vaarttaluds	3	0.7882	0.4347
*D. maculata*	Papendel 1	2	0.6254	0.2982
	Papendel 2	2	0.5991	0.3033
	Tiendeberg	2	0.5762	0.3082
	De Zegge	2	0.5942	0.3117
	Vrieselhof	2	0.5942	0.2848
	Vorsdonkbos	3	0.6580	0.4333
*D. majalis*	Buitengoor	3	0.8070	0.4159
	Aadgat	3	1.0515	0.4169
	Leiemeeersen	3	0.8284	0.4317
	Malendriesbeekvallei	6	1.1485	0.8143
	Revogne	5	0.8841	0.7292
	Snoekengracht	3	0.8197	0.4386
*D. praetermissa*	De Fonteintjes	6	1.1485	0.8139
	Gavers	2	0.7581	0.2943
	Warandeduinen	2	0.7751	0.3040

### Molecular Assessment of the Mycorrhizal Community

The fungal partners of orchids can be accurately identified directly from orchid protocorms, roots, tubers and rhizomes by various methods [7]. Here, we used DNA array technologies [31], [32] to detect and identify the fungi association with the sampled *Dactylorhiza* species. Prior to analysis, root fragments were cut in pieces of about 1 cm and after microscopic inspection of mycorrhizal colonization 0.5 g of mycorrhizal root pieces was used for DNA extraction using the UltraClean Plant DNA Isolation Kit as described by the manufacturer (Mo Bio Laboratories Inc., Solana Beach, CA, USA), and 10 times diluted afterwards. The mycorrhizal community associated with the roots was assessed as described previously [19], [31], [32]. First, clone libraries of fungal internal transcribed spacer (ITS) regions were constructed and sequenced for five randomly selected individuals per species. As a result, a total of 25 clone libraries was analyzed (representing ∼20% of the sampled individuals).

**Figure 3:**
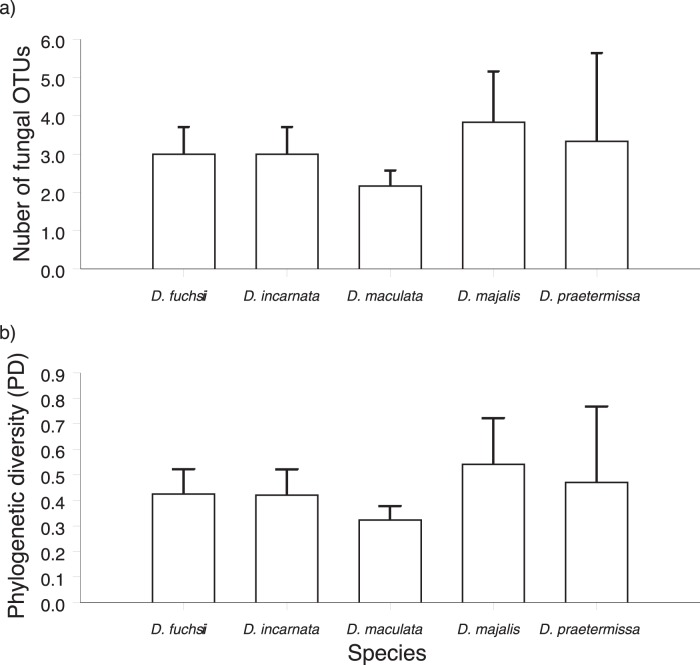
Number of fungal operational taxonomic units (OTUs) per population and phylogenetic diversity (PD) corrected for species richness in five *Dactylorhiza* species.

**Figure 4:**
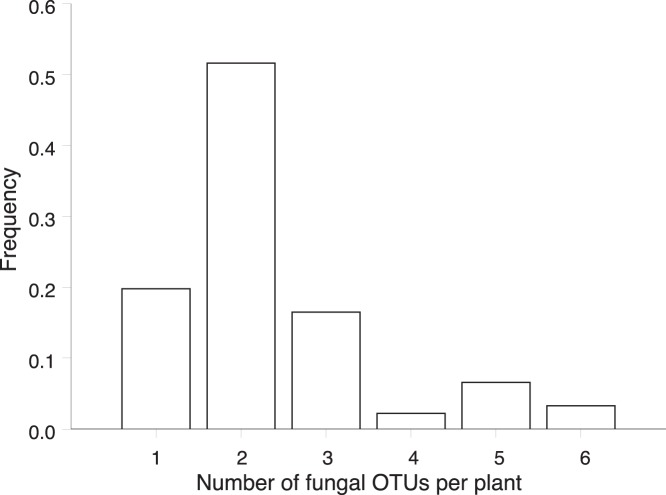
Frequency distribution of the number of fungal operational taxonomic units (OTUs) per individual plant across five *Dactylorhiza* species sampled in Belgium.

Clone libraries were constructed following PCR amplification with ITS1-OF and ITS4-Tul [33]. In a preliminary phase of this study, the performance of multiple primer pairs targeting different taxonomical levels, including the universal primer pair ITS1/ITS4 [34], the broad-spectrum basidiomycete primers ITS1-OF/ITS4-OF [33] and the Tulasnellaceae specific primers ITS1-OF/ITS4-Tul, was evaluated on a number of *Dactylorhiza* samples by a PCR screen and subsequent gel electrophoresis. ITS1-OF and ITS4-Tul turned out to be the most efficient primers for these samples as this primer pair gave the most consistent amplification with high yields. In addition, in contrast to the other two primer pairs, no amplification of plant sequences was observed using ITS1-OF and ITS4-Tul. ITS4-Tul is known to work well for core species of the genus *Tulasnella*, representing the major genus within orchid mycorrhizas [28], [33]. As such, this primer has been used widely to study orchid mycorrhizas [16], [20], [35]–[39], and has also been used to screen ectomycorrhizas for the presence and diversity of *Tulasnella* species [40].

**Figure 5:**
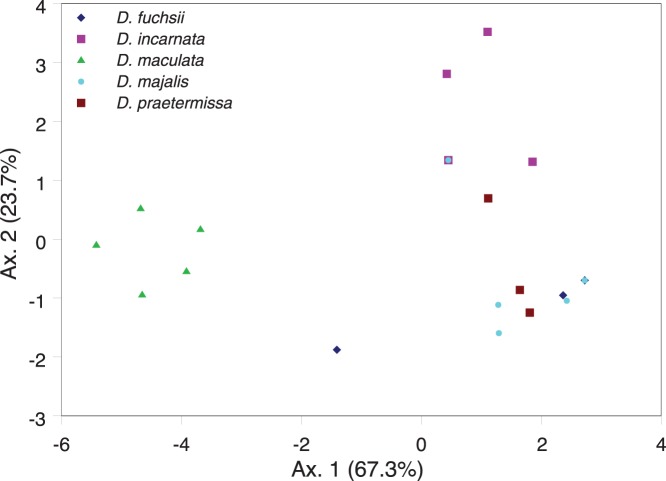
Discriminant analysis (DA) plots comparing mycorrhizal spectra in five *Dactylorhiza* species sampled in 26 populations in Belgium.

Out of each library, 96 clones were randomly picked and sequenced using the M13 forward primer. Subsequently, DNA sequences from the complete data set were aligned using the MEGA4 software package [41] followed by manual editing. Conserved sequence motifs were identified in the regions flanking each ITS sequence and the sequences were cut to these motifs. The sequences were further analyzed by the software package Mothur [42] and grouped into operational taxonomic units (OTUs), based on a conservative similarity threshold of 95%. This cutoff value was chosen as ITS sequences in *Tulasnella* are known to evolve rapidly [39] and, consequently, higher similarity cutoffs may overestimate fungal diversity. In order to identify the different OTUs, representative sequences for each OTU were queried against GenBank using BLAST. For each OTU five sequences have been deposited in GenBank (Accession numbers JX024729-JX024738). Because a subset of 25 individuals was used for clone library analyses, this may lead to underestimation of fungal diversity as rare fungi may be overlooked. To assess the magnitude of this effect, total species diversity as well as completeness of the sampling was assessed using rarefaction analyses [43].

Next, a DNA array was developed for the simultaneous detection and identification of the different OTUs found by the clone library analysis. For each OTU, six OTU-specific detector oligonucleotides were designed as described previously [31], [44] (Table S1). In order to enhance the accuracy of identification, oligonucleotides were derived from different regions in the ITS sequence. In addition to these oligonucleotides, a nonspecific oligonucleotide (Uni1) and a digoxigenin-labeled control oligonucleotide (Dig1) [44] (Table S1) were used as a confirmation for proper hybridization and detection, respectively. DNA array production, hybridization, washing, detection, and data analysis were performed as described previously [31], [32]. Hybridization was performed using digoxigenin-labeled amplicons generated using ITS1-OF and ITS4-Tul from all investigated plant individuals. All hybridizations were performed at least twice with similar results.

### Data Analysis

In order to assess the phylogenetic relationships among the observed fungi, a phylogenetic tree was created. To this end, the subtree A generated by [45], representing ITS sequences from tulasnelloid fungi from different terrestrial and epiphytic orchids belonging to other families of *Orchidaceae* and non-orchid species from different continents and environments was enriched with sequences from our own study. Sequences were aligned using the program ClustalX2 [46] with default conditions for gap opening and gap extension penalty, and adjusted manually. Phylogenetic reconstruction was performed using the Bayesian Markov Chain Monte Carlo (MCMC) inference (BI) method using the program MrBayes v 3.1.2 [47]. The substitution model (GTR +G) suggested as best-fit to the data under the corrected Akaike information criterion (AIC) was estimated using the program Kakusan 4 for Windows [48]. Two simultaneous, independent runs were performed for over 5 000 000 generations starting from random trees. Trees were sampled every 500 generations, resulting in a total of 10 001 trees from which the first 2 500 (25%) were discarded as the burn-in phase. A 50% majority rule consensus tree (Fig. 1) was calculated based on the remaining sampled trees, enabling the use of Bayesian Posterior Probabilities (BPP) as node support.

For each population, mycorrhizal specificity was determined by counting the number of fungal OTUs detected in each population. Additionally, we also calculated phylogenic diversity (PD) as ‘the minimum total length of all the phylogenetic branches required to span a given set of taxa on the phylogenetic tree’ [49]. Larger PD values can be expected to correspond to greater expected diversity. However, because PD values depend on the number of fungal taxa detected within a population, we also calculated PD values that correct for differences in sample size. PD values were assessed for each population using the R package Picante [50]. We used a one-way ANOVA (or its non-parametric alternative in case assumptions of normality were violated) to investigate whether the number of fungal OTUs and PD values differed significantly between species. To investigate whether the number of fungal OTUs observed per individual plant differed between species, a mixed model ANOVA was used with species as fixed factor and population as a random factor. We also tested the hypothesis that orchid rarity was related to mycorrhizal specificity by relating the number of grid cells occupied by each species to the total number of OTUs and PD values using a Spearman rank correlation.

Finally, to visualize the variation in fungal associations between populations within species and between species, a presence/absence matrix of all OTUs was created and used in discriminant analysis (DA). The analysis was performed using the discriminant function in SPSS 16.0 (SPSS).

## Results

In all plants investigated, signs of mycorrhizal colonization were observed. In total, the clone library analysis resulted in 1334 ITS sequences from tulasnelloid fungi. Based on a 95% cut-off value of similarity between sequences, 10 different tulasnelloid OTUs were distinguished (Table 1). BLAST analysis revealed relatively high sequence homology percentages with GenBank sequences, ranging from 94 to 100%. Out of the 10 observed OTUs, three OTUs appeared to be closely related to taxonomically identified members in the genus *Tulasnella*, including *T. calospora* (OTU8 and OTU10; both 96% sequence homology) and *T. irregularis* (OTU4; 97% homology). The sequences of the other seven OTUs showed highest homology with those from unidentified *Tulasnella* species. These originated from other plant species such as *Aneura pinguis* and *Riccardia multifida* (Table 1). Rarefaction analysis showed that the curve quickly reached an asymptote for the analyzed sequences (Fig. S2). Therefore, we decided that no further clone library analysis of the remaining root samples was necessary as a basis for the DNA array development as it was unlikely that new OTUs would be detected.

As expected, OTU sequences were scattered throughout the phylogenetic tree among fungal partners that have been described earlier in other terrestrial and epiphytic orchids (Fig. 1). The tree was rooted with sequences of the sister group B (*Tulasnella tomaculum* and *Tulasnella eichleriana*) [45]. Clusters comprising OTUs found in this study can be divided into three clades, called ‘TUL A’, ‘TUL B’ and ‘TUL C’. OTU1 and OTU4 turned out to be the most distantly related to the rest of the OTUs, and were included into two separate groups (TUL B and TUL C). The rest of OTU sequences grouped together into one major clade (TUL A). OTU8 and OTU10 were positioned in the same clade as *T. calospora* and OTU4 fell in the same clade as *T. irregularis*, which is comparable to the BLAST results (Table 1). OTU6 was closely related to a previously described fungal partner of *D. incarnata*.

Most OTUs were found in more than one *Dactylorhiza* species, and some of them were common and widespread (e.g. OTU1 and OTU9 were detected in 18 and 17 populations, respectively). OTU3, on the other hand, was only observed in one population. At the species level, five different OTUs were found in roots of *D. fuchsii,* six in *D. incarnata*, *D. maculata* and *D. praetermissa*, whereas nine OTUs were detected in *D. majalis* (Fig. 2). There was no significant (*P*>0.05) relationship between orchid rarity and the number of OTUs orchids associated with. All OTUs were nearly evenly spread across the studied orchid species. OTU9 was the most frequent OTU, occurring in about 27% of all samples. The OTUs with the lowest frequency were OTU2 and OTU3, occurring in 3 and 2% of all samples, respectively (Fig. 2). Both OTUs were detected only in one species. The most frequently occurring OTU (OTU9) was found in roots of all species. For *D. fuchsii* and *D. incarnata* it was the most common OTU (44% and 40% of occurrences, respectively). For *D. maculata* more than 32% of fungi found belonged to OTU5. In none of the species was an OTU found with a frequency >50% (Fig. 2), indicating that fungal species were relatively evenly distributed among individuals and populations.

The number of fungal OTUs that were detected within a population varied between two and six (average: 3.04) (Table 2). There was, however, no significant difference in the number of OTUs detected in a population between the five species (Kruskal Wallis χ^2^ = 5.78, *P* = 0.22) (Fig. 3a). PD values varied between 0.58 and 1.15 (Table 2), and were significantly different between species (Kruskal-Wallis χ^2^ = 14.08, *P* = 0.002). However, when correcting for differences in fungal richness, no significant differences between species were observed (χ^2^ = 8.70, *P* = 0.07) (Fig. 3b). Most plants also associated with more than one fungal partner at the same time (Fig. 4). About 50% of all plants associated with two different OTUs, and in nine individuals 5 or 6 different OTUs were observed on a single plant. Moreover, there was a significant difference (*F*_4,81.1_ = 8.52, *P*<0.001) in the number of fungal associates detected within a single individual between species. On average, less than two fungal OTUs were observed in individuals of *D. maculata*, whereas in *D. majalis* and *D. praetermissa* on average >3 OTUs per individual were detected.

Results of the Discriminant Analysis (DA) showed that in *D. incarnata, D. majalis* and *D. praetermissa* populations clustered together, while clustering was less pronounced for populations of *D. fuchsii* and *D. maculata* (Fig. 5). Populations of *D. maculata* were clearly separated from the other species, whereas there was less clear separation for the other species. However, along the second axis populations of *D. incarnata* were clearly separated from the remaining three species (*D. majalis*, *D. praetermissa* and *D. fuchsii*), which showed no clear segregation along either of the two axes.

## Discussion

### Mycorrhizal Partners

Using the primer set ITS1-OF/ITS4-Tul and a threshold value of 95% ITS sequence similarity we found ten different fungal OTUs associating with the five investigated *Dactylorhiza* species, all of which were related to members of the Tulasnellaceae. These results are consistent with previous reports stating that representatives of the genus *Tulasnella* are common associates found in the mycorrhizal flora of orchids [20], [36], [38], [39]. *Tulasnella* is a very common symbiont of terrestrial orchids and sequences related to the ones observed here have been observed in other European orchid genera, including *Anacamptis*, *Ophrys*, *Orchis* and *Serapias* [19], [45]. Although the use of a single primer pair may have underestimated total fungal diversity in these species, the performance of multiple primer pairs targeting different taxonomical levels was evaluated in a preliminary phase of this study. The results showed that the primer combination ITS1-OF and ITS4-Tul turned out to be the most efficient primer pair for these samples as it gave the most consistent amplification with high yields. In addition, no amplification of plant sequences was observed using this primer pair. Rarefaction analysis further showed that with a 95% threshold value the fungal community associating with the investigated species was accurately described, as the rarefaction curve clearly reached an asymptote.

Currently, knowledge about mycorrhizal associations in the genus *Dactylorhiza* is fragmentary, and there are no other studies that have systematically compared mycorrhizal associations between different species in the genus. The few studies published so far also found that species of the genus *Dactylorhiza* commonly associate with fungal taxa related to *Tulasnella*. In *D. majalis*, ITS sequences of symbionts found in roots fell into two main clades: one of the genus *Tulasnella* and a second one of distantly related *Laccaria* [27]. All 10 OTUs described here turned out to be moderately distant from the fungal partners described for *D. majalis* in Denmark [27]. OTU6 was strongly related to fungi that have been described for *D. incarnata* investigated in Hungary. The results are also consistent with observations in the related species *Gymnadenia conopsea*, which also consistently associated with representatives of the Tulasnellaceae or Ceratobasidiaceae [18]. Plants of *Dactylorhiza baltica*, on the other hand, associated exclusively with *Ceratobasidium albasitensis* [28].

### Multiple Fungal Associations

We showed that in 80% of all studied individuals orchids associated with more than one fungal OTU at the same time and in some individuals more than five different OTUs were detected simultaneously (Fig. 4). These results correspond to findings in the genus *Orchis*, in which multiple associations were also common [31], [32]. Multiple fungal associations were also found in several orchid species that grow in the understory of forests [20], [23] and in tropical myco-heterotrophic orchid species, in which sometimes more than five different fungal partners were observed on a single orchid species [26], [51]. On the other hand, adult plants never contained more than one fungal partner in the woodland orchid *Goodyera pubescens* [24]. Similarly, protocorms and adults of *Liparis liliifolia* were unable to associate with multiple fungi [24]. This raises the intriguing question whether different mycorrhizal partners have a similar function towards the plant. One possibility is that associating with multiple fungi may increase nutrient uptake by the orchid, as different fungal lineages are likely to have access to different nutrient resources [6], [52]. Associating with several fungal partners at the same time would also make switching from fungal partners unnecessary, a phenomenon that has been observed during stress conditions [24], and would thus decrease the chance to loose a fungal partner when shifting from one fungal OTU to another. However, it might also be possible that not all detected fungi are functioning as true mycorrhiza, as the identity of the fungi was determined by direct sequencing and their functionality was not tested experimentally.

### Fungal Specificity and Rarity

All investigated *Dactylorhiza* species associated with several common Tulasnella fungi from at least three different clades with low sequence similarity (<80%). In each investigated population, at least two different fungal OTUs were observed, and in two populations six different OTUs were found. These results largely correspond with data observed in the related genus *Orchis*. In this genus, species also associated with a large number of widely distributed fungal OTUs, and most OTUs were shared among species [19], [32]. Species of the Australian genera *Chiloglottis* and *Drakaea*, on the other hand, associated with a very narrow (less than 1% sequence dissimilarity) monophyletic *Tulasnella* clade [13], [53]. Although the exact reasons for this difference are not known, high mycorrhizal specificity could be related to landscape history [13]. Whereas *Drakaea* and *Chiloglottis* species occur in relatively old and stable landscapes, allowing specialization on one or a narrow clade of mycorrhiza(s) [13], the relatively young and highly disturbed landscapes in Europe may have favored generalist mycorrhizal associations.

Some fungal OTUs were widely distributed in the study region and occurred in more than 50% of the sampled populations. The occurrence of several fungal OTUs at multiple sites in multiple habitats suggests that suitable mycorhizal fungi are common within the study region and would not limit the formation of new populations. However, it should be noted that the diversity of fungi associating with mature orchids not necessarily reflects the fungi that are able to support seed germination [20]–[23]. In *Platanthera leucophaea*, for example, adults associated with a relatively wide range of *Ceratobasidium* spp., only one of which was shown to support seed germination [22]. Similarly, in *Tipularia discolor* plants associated with a very wide range of fungi, yet only two taxa supported seed germination [23]. Nonetheless, recent seed introduction experiments have shown that seeds of *D. fuchsii* and *D. praetermissa* germinated and developed into a protocorm in areas where the species were absent (De hert et al, unpublished manuscript), suggesting that mycorrhizal fungi are not the driving factor determining rarity in the investigated species. This is also in line with our observation that the distribution of the investigated *Dactylorhiza* species was not significantly related to the number of OTUs they associated with.

### Conclusion

In conclusion, analysis of mycorrhizal associations in five *Dactylorhiza* species sampled from a large number of populations in Belgium indicated that the investigated species associated with a wide range of Tulasnella fungi, some of which were common and widespread. Future studies should focus on other *Dactylorhiza* species that occur in other environmental conditions to elucidate the nature and specificity of mycorrhizal associations in this genus and to investigate whether mycorrhizal associations bear some phylogenetic imprint. Also, the mechanism behind sharing of multiple fungal partners is poorly understood. Because it is likely that different fungi play a different role in acquiring resources, more detailed investigations of the physiological mechanisms underpinning nutrient acquisition and sharing of fungal partners are needed [54]–[55].

## Supporting Information

Figure S1 **Location of sample sites of mycorrhizal fungi for DNA sequencing in five** ***Dactylorhiza*** **species in Belgium.**
(PDF)

Figure S2 **Rarefaction analysis performed on the internal transcribed spacer (ITS) sequence data obtained from the clone libraries for five** ***Dactylorhiza*** **species (1334 sequences), using a 95% sequence similarity threshold value.**
(PDF)

Table S1 **Detector oligonucleotides used in this study to detect ten different operational taxonomic units (OTUs) associated with five** ***Dactylorhiza*** **species sampled in Belgium.**
(PDF)

## References

[1] Govaerts R, Pfahl J, Campacci MA, Holland Baptista D, Tigges H, et al. (2011) World Checklist of *Orchidaceae* The Board of Trustees of the Royal Botanic Gardens, Kew. Available: http://www.kew.org/wcsp/Accessed 2011 Sept 15.

[2] Jacquemyn H, Brys R, Hermy M, Willems JH (2005) Does nectar reward affect rarity and extinction probabilities of orchid species? An assessment using historical records from Belgium and the Netherlands. Biological Conservation 121: 257–263.

[3] Kull T, Hutchings MJ (2006) A comperative analysis of decline in the distribution ranges of orchid species in Estonia and the United Kingdom. Biological Conservation 129: 31–39.

[4] Swarts NS, Dixon KW (2009) Terrestrial orchid conservation in the age of extinction. Annals of Botany 104: 543–556. 19218582 10.1093/aob/mcp025 PMC2720663

[5] Waterman RJ, Bidartondo MI (2008) Deception above, deception below: linking pollination and mycorrhizal biology of orchids. Journal of Experimental Botany 59: 1085–1096. 18316318 10.1093/jxb/erm366

[6] Waterman RJ, Bidartondo MI, Stofberg J, Combs JK, Gebauer G, et al (2011) The effects of above- and belowground mutualisms on orchid speciation and coexistence. American Naturalist 177: E54–E68. 10.1086/657955 21460551

[7] Dearnaley JDW, Martos F, Selosse M-A (2012) Orchid mycorrhizas: molecular ecology, physiology, evolution and conservation aspects. The Mycota 15: in press.

[8] Smith SE, Read DJ (2008) Mycorrhizal symbiosis. 3^rd^ ed. Sydney: Academic Press.

[9] Rasmussen HN, Rasmussen FN (2009) Orchid mycorrhiza: implications of a mycophagous life cycle. Oikos 118: 334–345.

[10] Bonnardeaux Y, Brundrett M, Batty A, Dixon K, Koch J, et al (2007) Diversity of mycorrhizal fungi of terrestrial orchids: compatibility webs, brief encounters, lasting relationships and alien invasions. Mycological Research 111: 51–61. 17289365 10.1016/j.mycres.2006.11.006

[11] Dearnaley JDW (2007) Further advances in orchid mycorrhizal research. Mycorrhiza 17: 475–486. 17582535 10.1007/s00572-007-0138-1

[12] Swarts NS, Sinclair EA, Francis A, Dixon KW (2010) Ecological specialization in mycorrhizal symbiosis leads to rarity in an endangered orchid. Molecular Ecology 19: 3226–3242. 20618899 10.1111/j.1365-294X.2010.04736.x

[13] Phillips RD, Barrett MD, Dixon KW, Hopper SD (2011) Do mycorrhizal symbioses cause rarity in orchids? Journal of Ecology 99: 858–869.

[14] Otero JT, Flanagan NS (2006) Orchid diversity – beyond deception. Trends in Ecology and Evolution 21: 64–65. 16701475 10.1016/j.tree.2005.11.016

[15] Taylor DL, Bruns TD (1997) Independent, specialized invasion of ectomycorrhizal mutualism by two non-photosynthetic orchids. Proceedings of the National Academy of Sciences of the United States of America 94: 5410–5415. 10.1073/pnas.94.9.4510 PMC20753 9114020

[16] Shefferson RP, Taylor DL, Weiss M, Garnica S, McCormick MK, et al (2007) The evolutionary history of mycorrhizal specificity among lady’s slipper orchids. Evolution 61: 1380–1390. 17542847 10.1111/j.1558-5646.2007.00112.x

[17] Ogura-Tsujita Y, Yukawa T (2008) High mycorrhizal specificity in a widespread mycoheterotrophic plant, *Eulophia zollingeri* (Orchidaceae). American Journal of Botany 95: 93–97. 21632319 10.3732/ajb.95.1.93

[18] Stark C, Babik W, Durka W (2009) Fungi from the roots of the common terrestrial orchid *Gymnadenia conopsea* . Mycological Research 113: 952–959. 19486943 10.1016/j.mycres.2009.05.002

[19] Jacquemyn H, Merckx V, Brys R, Tyteca D, Cammue BPA, et al (2011) Analysis of network architecture reveals phylogenetic constraints on mycorrhizal specificity in the genus *Orchis* (Orchidaceae). New Phytologist 192: 518–528. 21668874 10.1111/j.1469-8137.2011.03796.x

[20] Bidartondo MI, Burghardt B, Gebauer G, Burns TD, Read DJ (2004) Changing partners in the dark: isotopic and molecular evidence of ectomycorrhizal liaisons between forest orchids and trees. Proceedings of the Royal Society of London Series B - Biological Sciences 271: 1799–1806. 15315895 10.1098/rspb.2004.2807 PMC1691795

[21] Bidartondo MI, Read DJ (2008) Fungal specificity bottlenecks during orchid germination and development. Molecular Ecology 17: 3707–3716. 18627452 10.1111/j.1365-294X.2008.03848.x

[22] Zettler LW, Piskin KA (2012) Mycorrhizal fungi from protocorms, seedlings and mature plants of the Eastern Prairie Fringed Orchid, *Platanthera leucophaea* (Nutt.) Lindley: A comprehensive list to augment conservation. American Midland Naturalist 166: 29–39.

[23] McCormick MK, Whigham DF, O’Neill J (2004) Mycorrhizal diversity in photosynthetic terrestrial orchids. New Phytologist 163: 425–438. 10.1111/j.1469-8137.2004.01114.x 33873625

[24] McCormick MK, Whigham DF, Sloan D, O’Malley K, Hodkinson B (2006) Orchid-fungus fidelity: a marriage meant to last? Ecology 87: 903–911. 16676534 10.1890/0012-9658(2006)87[903:ofammt]2.0.co;2

[25] McKendrick SL, Leake JR, Taylor DL, Read DJ (2002) Symbiotic germination and development of the myco-heterotrophic orchid *Neottia nidus-avis* in nature and its requirement for locally distributed *Sebacina* spp. New Phytologist 154: 233–247.

[26] Martos F, Dulormne M, Pailler T, Bonfante P, Faccio A, et al (2009) Independent recruitment of saprotrophic fungi as mycorrhizal partners by tropical achlorophyllous orchids. New Phytologist 184: 668–681. 19694964 10.1111/j.1469-8137.2009.02987.x

[27] Kristiansen KA, Taylor DL, Kjoller R, Rasmussen HN, Rosendahl S (2001) Identification of mycorrhizal fungi from single pelotons of *Dactylorhiza majalis* (Orchidaceae) using single-strand conformation polymorphism and mitochondrial ribosomal large subunit DNA sequences. Molecular Ecology 10: 2089–2093. 11555252 10.1046/j.0962-1083.2001.01324.x

[28] Shefferson RP, Kull T, Tali K (2008) Mycorrhizal interactions of orchids colonizing Estonian mine tailings hills. American Journal of Botany 95: 156–164. 21632341 10.3732/ajb.95.2.156

[29] Devos N, Raspé O, Oh SH, Tyteca D, Jacquemart AL (2006) The evolution of *Dactylorhiza* (Orchidaceae) allotetraploid complex: Insights from nrDNA sequences and cpDNA PCR-RFLP data. Molecular Phylogenetics and Evolution 38: 767–778. 16439164 10.1016/j.ympev.2005.11.013

[30] Rasmussen HN (1995) Terrestrial orchids: from seed to mycotrophic plant. New York: Cambridge University Press.

[31] Lievens B, van Kerckhove S, Justé A, Cammue BPA, Honnay O, et al (2010) From extensive clone libraries to comprehensive DNA arrays for efficient and simultaneous detection and indentification of orchid mycorrhizal fungi. Journal of Microbiological Methods 80: 76–85. 19914306 10.1016/j.mimet.2009.11.004

[32] Jacquemyn H, Honnay O, Cammue BPA, Brys R, Lievens B (2010) Low specificity and nested subset stracture characterize mycorrhizal associations in five closely related species of the genus *Orchis* . Molecular Ecology 19: 4086–4095. 20735736 10.1111/j.1365-294X.2010.04785.x

[33] Taylor DL, McCormick MK (2008) Internal transcribed spacer primers and sequences for improved characterization of basidiomycetous orchid mycorrhizas. New Phytologist 177: 1020–1033. 18086221 10.1111/j.1469-8137.2007.02320.x

[34] White TJ, Bruns TD, Lee SB, Taylor JW (1990) Amplification and direct sequencing of fungal ribosomal RNA genes for phylogenetics. In: Innis MA, Gelfand H, Sninsky JS, White TJ, editors. PCR-Protocols and Applications-A laboratory Manual. New York: Academic Press. 315–322.

[35] Julou T, Burghardt B, Gebauer G, Berveiller D, Damesin C, et al (2005) Mixotrophy in orchids : insights from a comperative study of green individuals and nonphotosynthetic individuals of *Cephalanthera damasonium* . New Phytologist 166: 639–653. 15819926 10.1111/j.1469-8137.2005.01364.x

[36] Shefferson RP, Weiß M, Kull T, Taylor DL (2005) High specificity generally characterizes mycorrhizal association in rare lady’s slipper orchids, genus *Cypripedium* . Molecular Ecology 14: 613–625. 15660950 10.1111/j.1365-294X.2005.02424.x

[37] Abadie JC, Püttsepp Ü, Gebauer G, Faccio A, Bonfante P, et al (2006) *Cephalanthera longifolia* (Neottieae, Orchidaceae) is mixotrophic: a comparative study between green and non-photosynthetic individuals. Canadian Journal of Botany 84: 1462–1477.

[38] Girlanda M, Selosse MA, Cafasso D, Brilli F, Delfine S, et al (2006) Inefficient photosynthesis in the Mediterranean orchid *Limodorum abortivum* is mirrored by specific association to ectomycorrhizal Russulaceae. Molecular Ecology 15: 491–504. 16448415 10.1111/j.1365-294X.2005.02770.x

[39] Suarez JP, Weiß M, Abele A, Garnica S, Oberwinkler F, et al (2006) Diverse tulasnelloid fungi from mycorrhizas with epiphytic orchids in an Andean cloud forest. Mycological Research 110: 1257–1270. 17081740 10.1016/j.mycres.2006.08.004

[40] Bidartondo MI, Bruns TD, Weiß M, Sergio C, Read DJ (2003) Specialized cheating of the ectomycorrhizal symbiosis by an epiparasitic liverwort. Proceedings of the Royal Society of London Series B - Biological Sciences 270: 835–842. 12737662 10.1098/rspb.2002.2299 PMC1691308

[41] Tamura K, Dudley J, Nei M, Kumar S (2007) Mega4: Molecular Evolutionary Genetics Analysis (MEGA) Software, Version 4.0. Molecular Biology and Evolution 24: 1596–1599. 17488738 10.1093/molbev/msm092

[42] Schloss PD, Westcott SL, Ryabin T, Hall JR, Hartmann M, et al (2009) Introducing mothur: Open-source, platform-independent, community-supported software for describing and comparing microbial communities. Applied and Environmental Microbiology 75: 37–41. 10.1128/AEM.01541-09 PMC2786419 19801464

[43] Hurlbert SH (1971) The nonconcept of species diversity: a critique and alternative parameters. Ecology 52: 577–586. 28973811 10.2307/1934145

[44] Lievens B, Brouwer M, Vanachter ACRC, Lévesque CA, Cammue BPA, et al (2003) Design and development of a DNA array for rapid detection and identification of multiple tomato vascular wilt pathogens. FEMS Microbiology Letters 223: 113–122. 12799009 10.1016/S0378-1097(03)00352-5

[45] Girlanda M, Segreto R, Cafasso D, Liebel HT, Rodda M, et al (2011) Photosynthetic Mediterranean meadow orchids feature partial mycoheterotrophy and specific mycorrhizal associations. American Journal of Botany 98: 1148–1163. 21712419 10.3732/ajb.1000486

[46] Larkin MA, Blackshields G, Brown NP, Chenna R, McGettigan PA, et al (2007) ClustalW and ClustalX version 2. Bioinformatics 23: 2947–2948. 17846036 10.1093/bioinformatics/btm404

[47] Huelsenbeck JP, Ronquist F (2001) MrBayes: Bayesian inference of phylogenetic trees. Bioinformatics 17: 754–755. 11524383 10.1093/bioinformatics/17.8.754

[48] Tanabe AS (2011) Kakusan4 and Aminosan: two programs for comparing nonpartitioned, proportional, and separate models for combined molecular phylogenetic analyses of multilocus sequence data. Molecular Ecology Resources 11: 914–921. 21592310 10.1111/j.1755-0998.2011.03021.x

[49] Faith DP (1992) Conservation evaluation and phylogenetic diversity. Biological Conservation 61: 1–10.

[50] Kembel SM, Cowan PD, Helmus MR, Cornwell WK, Morlon H, et al (2010) Picante: R tools for integrating phylogenies and ecology. Bioinformatics 26: 1463–1464. 20395285 10.1093/bioinformatics/btq166

[51] Roy M, Watthana S, Stier A, Richard F, Vessabutr S, et al (2009) Two mycoheterotrophic orchids from Thailand tropical dipterocarpacean forests associate with a broad diversity of ectomycorrhizal fungi. BMC Biology 7: 51. 19682351 10.1186/1741-7007-7-51 PMC2745373

[52] Leake JR, Cameron DD (2010) Physiological ecology of mycoheterotrophy. New Phytologist 185: 601–605. 20356334 10.1111/j.1469-8137.2009.03153.x

[53] Roche SA, Carter RJ, Peakall R, Smith LM, Whitehead MR, et al (2010) A narrow group of monophyletic *Tulasnella* (*Tulasnellaceae*) symbionts lineages are associated with multiple species of *Chiloglottis* (*Orchidaceae*): implications for orchid diversity. American Journal of Botany 97: 1313–1327. 21616884 10.3732/ajb.1000049

[54] Gebauer G, Meyer M (2003) ^15^N and ^13^C natural abundance of autotrophic and mycoheterotrophic orchids provides insight into nitrogen and carbon gain from fungal association. New Phytologist 160: 209–223. 10.1046/j.1469-8137.2003.00872.x 33873535

[55] Liebel HT, Bidartondo MI, Preiss K, Segreto R, Stöckel M, et al (2010) C and N stable isotope signatures reveal constraints to nutritional modes in orchids from the Mediterranean and Macronesia. American Journal of Botany 97: 903–912. 21622461 10.3732/ajb.0900354

