# Supplementary material for: Variation in Mycorrhizal Associations with Tulasnelloid Fungi among Populations of Five Dactylorhiza Species
Source: PLoS One. 2012 Aug 3;7(8):e42212. doi: 10.1371/journal.pone.0042212 (PMC3411701; doi:10.1371/journal.pone.0042212)

Fig. S1 Location of sample sites of mycorrhizal fungi for DNA sequencing in five *Dactylorhiza* species in Belgium.

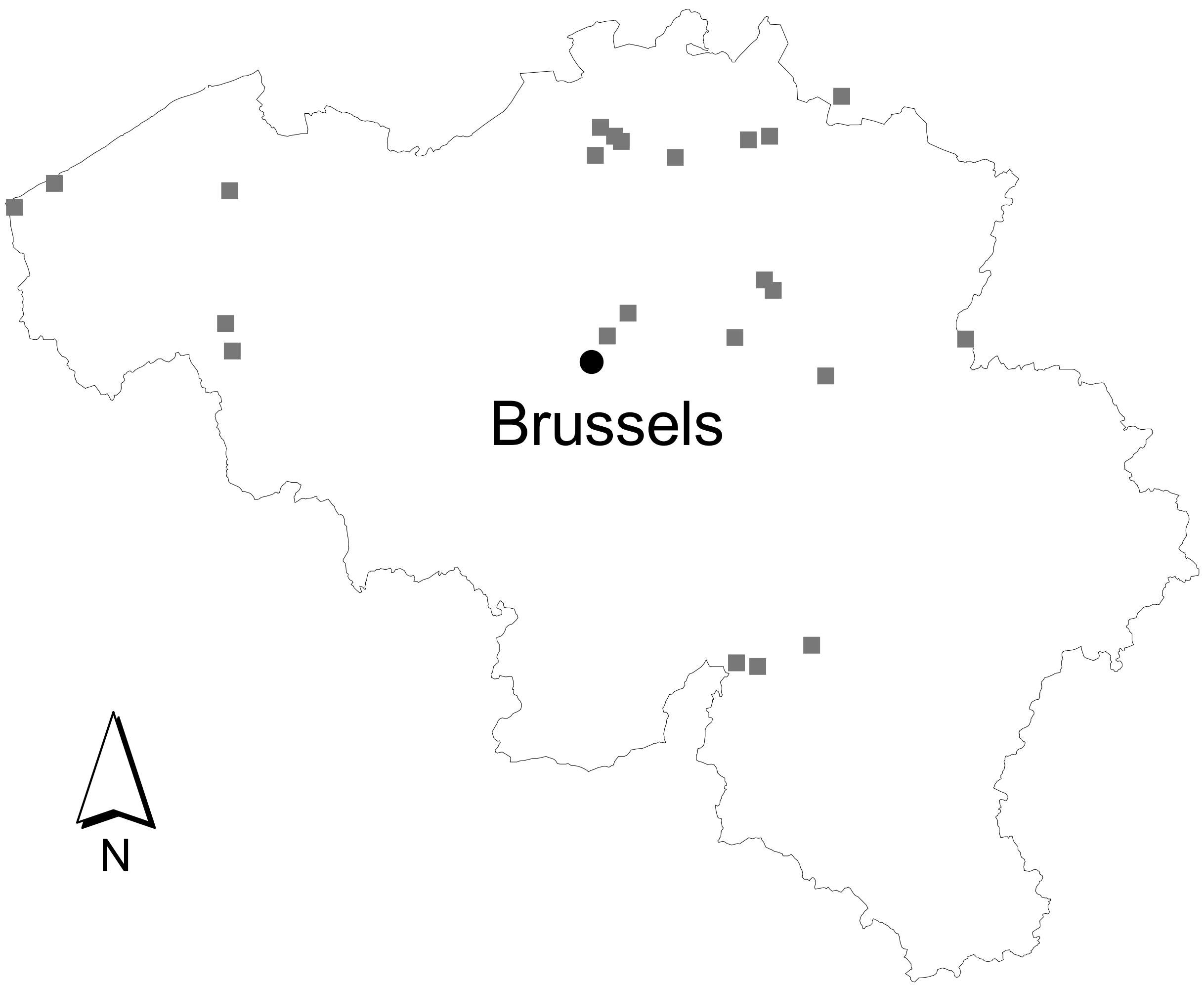

Supplement: Figure S1 — Location of sample sites of mycorrhizal fungi for DNA sequencing in five Dactylorhiza species in Belgium. (PDF) [file pone.0042212.s001.pdf]
