# Supplementary material for: Variation in Mycorrhizal Associations with Tulasnelloid Fungi among Populations of Five Dactylorhiza Species
Source: PLoS One. 2012 Aug 3;7(8):e42212. doi: 10.1371/journal.pone.0042212 (PMC3411701; doi:10.1371/journal.pone.0042212)

Fig. S2 Rarefaction analysis performed on the internal transcribed spacer (ITS) sequence data obtained from the clone libraries for five *Dactylorhiza* species (1334 sequences), using a 95% sequence similarity threshold value.

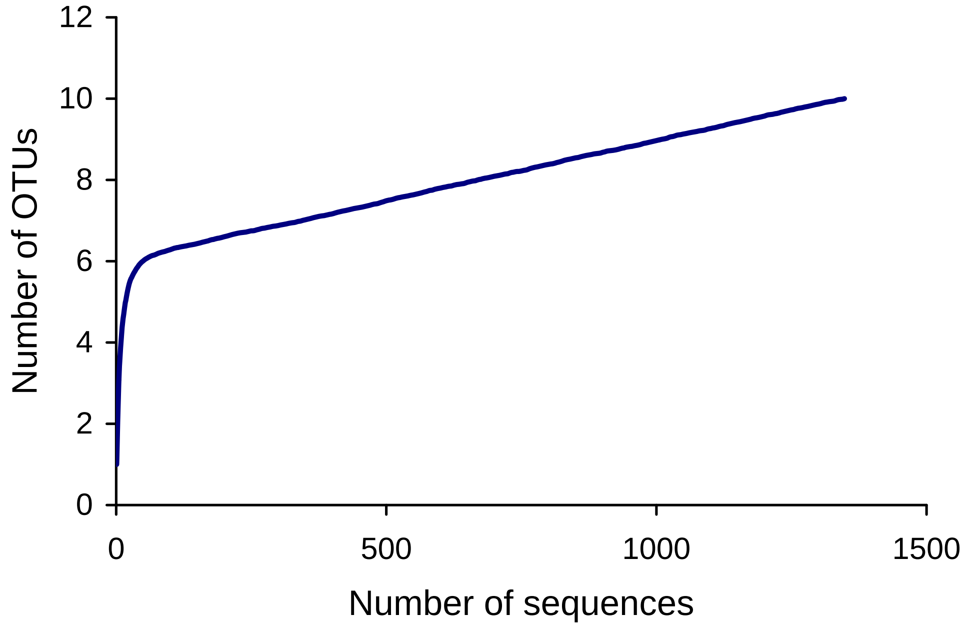

Supplement: Figure S2 — Rarefaction analysis performed on the internal transcribed spacer (ITS) sequence data obtained from the clone libraries for five Dactylorhiza species (1334 sequences), using a 95% sequence similarity threshold value. (PDF) [file pone.0042212.s002.pdf]
