# Supplementary material for: Variation in Mycorrhizal Associations with Tulasnelloid Fungi among Populations of Five Dactylorhiza Species
Source: PLoS One. 2012 Aug 3;7(8):e42212. doi: 10.1371/journal.pone.0042212 (PMC3411701; doi:10.1371/journal.pone.0042212)
Supplement: Table S1 — Detector oligonucleotides used in this study to detect ten different operational taxonomic units (OTUs) associated with five Dactylorhiza species sampled in Belgium. (PDF) [file pone.0042212.s003.pdf]

Table S1 Detector oligonucleotides used in this study to detect ten different operational taxonomic units (OTUs) associated with five *Dactylorhiza* species sampled in Belgium.

Target OTU	Name	Sequence (5'-3')
OTU1	OTU1'_1_1	TAATCGTCTATCGACGCACT
	OTU1'_1_2	CGTCTATCGACGCACTTGCT
	OTU1'_1_3	TCGTCTATCGACGCACTTGC
	OTU1'_3_1	CATTATTTCGTTCGCCGAG
	OTU1'_3_2	TTATTTCGTTCGCCGAGCC
	OTU1'_3_3	TTTCGTTCGCCGAGCCTCG
OTU2	OTU2'_2_1	CGAGTCTTGAGCTTGTGTCA
	OTU2'_2_2	ACCGAGTCTTGAGCTTGTGT
	OTU2'_2_3	AGTCTTGAGCTTGTGTCACC
	OTU2'_3_1	CAAACGGTACTACAACCCAT
	OTU2'_3_2	AACAAACGGTACTACAACCC
	OTU2'_3_3	ACGGTACTACAACCCATGAC
OTU3	OTU3'_2_2	AGGTAAAGCCGTCTCCCTG
	OTU3'_2_3	GAGGTAAAGCCGTCTCCCTG
	OTU3'_5_1	AGTCTTTTCATCGAGACCCG
	OTU3'_5_2	GTCTTTTCATCGAGACCCGA
	OTU3'_7_1	CACGTCTTACGTGGCGGTA
	OTU3'_7_3	ACGTCTTACGTGGCGGTACT
OTU4	OTU4'_3_2	TTTCGGTCCGTGATGCCCG
	OTU4'_4_1	AAGGGAAGAGGCCACGCCG
	OTU4'_4_2	GAAGGGAAGAGGCCACGCC
	OTU4'_6_2	AGGACGTGTCCGCAGCGAG
	OTU4'_6_3	CGCCGAAGCCCAGGACGTG
	OTU4'_7_2	AGACGCACCTTCACGGTGAA
OTU5	OTU5'_1_2	CTCTGTGTTACCTCTAAAAATT
	OTU5'_1_3	TCTGTGTTACCTCTAAAAATTT
	OTU5'_1_4	CCTCTAAAAATTTTTTCCGAG
	OTU5'_1_5	TCTAAAAATTTTTTCCGAGGC
	OTU5'_1_6	CCTCTAAAAATTTTTTTCCGAG
	OTU5'_1_7	TCTAAAAATTTTTTTCCGAGGC
OTU6	OTU6'_1_4	AGTCCCTTTGAGCTTGAGAC
	OTU6'_2_1	TGAAGCGGGACCCGCTCAA
	OTU6'_2_2	GTGAAGCGGGACCCGCTCA
	OTU6'_2_3	GAAGCGGGACCCGCTCAAT
	OTU6'_3_1	GTTAAACGGTCGCTT**C**CCTG
	OTU6'_3_3	GGTTAAACGGTCGCTT**C**CCT
OTU7	OTU7'_1_2	AGTCCGCCTACCAGCGGTA
	OTU7'_1_3	GTCCGCCTACCAGCGGTAC
	OTU7'_2_1	CTTTTCCGTCGTCCTTGGGA
	OTU7'_2_2	TTCCGTCGTCCTTGGGACA
	OTU7'_2_3	CCGTCGTCCTTGGGACATTA
	OTU7'_3_1	CCTCTAAAAAAATTTTTCCGAG
OTU8	OTU8'_1_1	TTAAATGGTCGCTGCTGTGTT
	OTU8'_1_2	AATGGTCGCTGCTGTGTTAC
	OTU8'_2_1	GTCCTTTCTGGGTTTGAGACG

	OTU8'_2_3	TTCTGGGTTTGAGACGTGCT
	OTU8'_3_1	GCGTCCTTTGCGATGTCGGT
	OTU8'_3_2	CGTCCTTTGCGATGTCGGTA
OTU9	OTU9'_1_2	TTTCTTCCGAGGCACACGTTA
	OTU9'_1_3	AATTTTCTTCCGAGGCACACG
	OTU9'_1_4	CGTGTGCCTCGGAAGAAAATT
	OTU9'_2_2	CTCTTCTGGATCGTGTTCTCTTA
	OTU9'_2_3	AACACGATCCAGAAGAGGAC
	OTU9'_3_2	AGCAGTACTACAACACATGACC
OTU10	OTU10'_1_2	TAACACAGCGACCGCTTAAC
	OTU10'_1_4	AGGTAACACAGCGACCGCTT
	OTU10'_2_1	GCTGTGTTACCTCTTTTCGAG
	OTU10'_2_2	CTGTGTTACCTCTTTTCGAGG
	OTU10'_4_1	GTCCTTTGGGACGGCGGTA
	OTU10'_4_2	TCCTTTGGGACGGCGGTAC
Universal	Uni1[a]	TCCTCCGCTTATTGATATGC
None	Dig1[a,b]	GTCCAGACAGGATCAGGATTG

[a] Lievens et al. (2003).

[b] 5′-end thiol and 3′-end digoxigenin-labeled reference oligonucleotide.
